# Effects of being uninsured or underinsured and living in extremely poor neighborhoods on colon cancer care and survival in California: historical cohort analysis, 1996—2011

**DOI:** 10.1186/1471-2458-12-897

**Published:** 2012-10-24

**Authors:** Kevin M Gorey, Isaac N Luginaah, Eric J Holowaty, Guangyong Zou, Caroline Hamm, Emma Bartfay, Sindu M Kanjeekal, Madhan K Balagurusamy, Sundus Haji-Jama, Frances C Wright

**Affiliations:** 1 School of Social Work, University of Windsor, 401 Sunset Avenue, Windsor, Ontario, N9B 3P4, Canada; 2 Department of Geography, University of Western Ontario, London, Ontario, Canada; 3 Dalla Lana School of Public Health, University of Toronto, Toronto, Ontario, Canada; 4 Department of Epidemiology and Biostatistics, and Robarts Research Institute, University of Western Ontario, London, Ontario, Canada; 5 Windsor Regional Cancer Center and School of Medicine and Dentistry, Department of Medicine, Division of General Internal Medicine, University of Western Ontario, London, Ontario, Canada; 6 Faculty of Health Sciences, University of Ontario Institute of Technology, Oshawa, Ontario, Canada; 7 Windsor Regional Cancer Center, Windsor, Ontario, Canada; 8 School of Social Work, University of Windsor, Windsor, Ontario, Canada; 9 School of Social Work, University of Windsor, Windsor, Ontario, Canada; 10 Division of General Surgery, Sunnybrook Health Sciences Center and cross appointed to the Departments of Surgery, and Health Policy, Management and Evaluation, University of Toronto, Toronto, Ontario, Canada

**Keywords:** Health insurance, Uninsured, Colon cancer care, Surgery, Chemotherapy, Wait times, Survival, Poverty, High poverty neighborhoods, Health care reform, California, United States

## Abstract

**Background:**

We examined the mediating effects of health insurance on poverty-colon cancer care and survival relationships and the moderating effects of poverty on health insurance-colon cancer care and survival relationships among women and men in California.

**Methods:**

We analyzed registry data for 3,291 women and 3,009 men diagnosed with colon cancer between 1996 and 2000 and followed until 2011 on lymph node investigation, stage at diagnosis, surgery, chemotherapy, wait times and survival. We obtained socioeconomic data for individual residences from the 2000 census to categorize the following neighborhoods: high poverty (30% or more poor), middle poverty (5-29% poor) and low poverty (less than 5% poor). Primary health insurers were Medicaid, Medicare, private or none.

**Results:**

Evidence of mediation was observed for women, but not for men. For women, the apparent effect of poverty disappeared in the presence of payer, and the effects of all forms of health insurance seemed strengthened. All were advantaged on 6-year survival compared to the uninsured: Medicaid (RR = 1.83), Medicare (RR = 1.92) and private (RR = 1.83). Evidence of moderation was also only observed for women. The effects of all forms of health insurance were stronger for women in low poverty neighborhoods: Medicaid (RR = 2.90), Medicare (RR = 2.91) and private (RR = 2.60). For men, only main effects of poverty and payers were observed, the advantaging effect of private insurance being largest. Across colon cancer care processes, Medicare seemed most instrumental for women, private payers for men.

**Conclusions:**

Health insurance substantially mediates the quality of colon cancer care and poverty seems to make the effects of being uninsured or underinsured even worse, especially among women in the United States. These findings are consistent with the theory that more facilitative social and economic capital is available in more affluent neighborhoods, where women with colon cancer may be better able to absorb the indirect and direct, but uncovered, costs of care.

## Background

A study of cancer survival in low-income areas of Toronto, Ontario, and Detroit, Michigan, during the 1980s found advantages among Canadians for common cancers including colon cancer [1]. The Canadian survival advantage was systematically replicated across diverse low-income Canadian and US contexts through the 1990s [2-8], culminating in a recent study of colon cancer treatment accessibility and survival in Toronto, Ontario, and San Francisco, California that followed its cohorts until 2006 [9]. In the United States, people with colon cancer who lived in low-income areas experienced less thorough lymph node evaluations and were less likely to receive indicated chemotherapies or to survive. More inclusive health insurance in Canada was advanced as the most plausible explanation.

All of these studies were ecological with respect to the measurement of socioeconomic status (SES). They typically used census tract data to define low-income neighborhoods where cancer patients lived, however, their lowest income areas only ranged from 10% to 20% poor. So they had limited power to study cancer care among “the truly disadvantaged” [10] who live, for example, in America’s poorest neighborhoods where 30% to 40% or more of the households have incomes below the poverty line [10-12]. Recent synthetic and exploratory analyses have suggested the potential great policy importance of such study [13,14] and studies of colon cancer care may be particularly instructive in such contexts for several reasons.

First, colon cancer is the second most frequent cause of cancer death in America and its prognosis can be excellent with early diagnosis and treatment [15]. Second, generally modest inverse associations between income and colon cancer survival have been consistently observed in the US [1,9,16-19]. Third, as colon cancer screens have begun to be implemented and as effective chemotherapies proliferated during the 1990s for stage III colon cancer and more recently for stage II disease [20-22], evidence has mounted that the best care, from initial screening, through diagnostic investigations to follow-up care, is more accessible to people with higher SES [9,22-29]. Fourth, various underinsured statuses have also been associated with less than optimum colon cancer care in America [22,23,30-36]. Similar to international studies, these US studies have for the most part had insufficient power to study the quality of colon cancer care among those at greatest risk of not receiving it; the poor and the uninsured. Their samples of such people have tended to be quite small or nonexistent. One naturally wonders if even larger colon cancer survival disadvantages would be observed, for example, among more extremely poor people living in America’s most impoverished places where health risks, including the risk of not having health insurance, are most prevalent.

### Combined effects of being underinsured and living in extremely poor communities

Our previous within-US and Canada-US comparative studies of cancer care have allowed us to develop the theory that SES-cancer care relationships are probably mediated by health insurance [9,13,37]. We think that it is also probable that within the multi-payer US health care system the effects of various payers (self, Medicaid, Medicare and/or various private insurance companies) interact with other socioeconomic resources, personal and community, in complex ways that have not yet been studied. In fact, thus far this field has primarily advanced knowledge about main effects, that is, hypotheses about the independent effects of SES or various payers have been tested alone. More complex hypotheses about mediation and moderation effects have not been formally tested.

Our SES-payer-cancer care mediation theory borrows constructs from sociology, medicine and public health, and aims principally to model care gaps in America’s most vulnerable communities. Its foundation is William Julius Wilson’s germinal work in 1960s Chicago’s high poverty neighborhoods (30% or more poor) and Paul Jargowsky and Mary Jo Bane’s construct validation of hundreds of extremely poor neighborhoods (40% or more poor) across America between 1970 and 1990 [10-12]. Together they described places of prevalent demographic vulnerability that are particularly distressed for their lack of social and economic capital. In addition to the poor, all of the following groups of people tend to be more concentrated there: ethnic and racial minority group members, recent immigrants, young adults without a high school diploma, single mothers, the unemployed and those who have withdrawn from the labor market, part-time service workers versus full time skilled workers or professionals, renters versus home owners, welfare recipients and the homeless. Medicaid and Medicare, the government’s, respective, mean-tested and entitlement, health care programs were specifically designed to mediate the effects of such concentrated impoverishment. But SES and various payers, including private ones, probably interact in other important ways across America’s economic divide.

A recent survey practically demonstrated how SES and health insurance status could interact in the lives of real people with colon cancer. It surveyed nearly 300 people with stage III colon cancer, most of whom were insured and found that more than a third of them (38%) suffered one or more significant financial hardships as a result of their treatment [38,39]. These included selling or refinancing their home, borrowing money from friends or family, experiencing an income decline of 20% or more, and prematurely withdrawing money from retirement accounts. Moreover, more than one of every ten of them reported coverage denials by their insurers and consequently one of every ten to fifteen of them was not able to adhere to their prescribed treatment regime. And perhaps not surprisingly, low-income households were much more likely to be so impacted. So it seems that the ultimate effectiveness of various health insurance programs may be significantly impacted or moderated by other available resources. And those most likely to be disadvantaged are the poor. It is they who will probably be least likely to pick up the indirect costs (time lost from work for numerous treatment visits, recuperation and perhaps travel) and direct, but uncovered costs (co-insurance and co-payments) of contemporary cancer care in America.

The objective of this study was to evaluate the mediating and moderating effects of SES and health insurance payer status on cancer care in extremely poor communities in the US. We hypothesized that the inverse poverty-colon cancer survival relationship would be mediated by payers (any payer would be better than being uninsured). We also hypothesized that the direct payer-colon cancer survival relationship would be moderated by poverty (all payers would be less effective in high poverty neighborhoods). Because gender is well known to be strongly associated with being poor and uninsured in the US [40,41] and our preliminary analyses suggested that survival disadvantages associated with poverty were greater for women than for men with colon cancer, we based our analyses upon the exploratory hypotheses that mediation and moderation effects would both be stronger among women. We then explored similar mediation and moderation hypotheses across the colon cancer care continuum: thoroughness of diagnostic investigation, stage of disease at diagnosis, receipt of and wait times for surgery and adjuvant chemotherapy, and receipt of palliative care.

## Methods

The sampling frame was the California cancer registry which monitors the most populous US state. It ascertains nearly all colon cancer cases with a nearly perfect rate of microscopic confirmation [42,43]. Registry data was obtained for 6,300 colon cancer cases diagnosed between 1996 and 2000 (International Classification of Diseases, Ninth Revision code 153 [44]) and followed until 2011. Cases were randomly selected from 3 place and 3 poverty strata: megalopolises with between 3 and 12 million residents (greater San Diego, the San Francisco Bay area and greater Los Angeles), smaller metropolitan areas with populations between 400,000 and 900,000 (Salinas, Modesto, Stockton, Bakersfield and Fresno), and small rural places with populations of less than 10,000 and population densities less than 400 per km^2^[45-47]. Overall, we sampled 15.4% of the colon cancer cases that were diagnosed in these places between 1996 and 2000. This study was powered to detect a difference of 5% in survival rates for women and men across 3 places and 3 poverty groups with 80% power at a 2-tailed significance level of 5% [48,49]. A 15% oversample was drawn to account for unstaged cases and other missing data.

### Variables

#### Poverty and payers

We first linked colon cancer patients to the US (2000) census by their residential census tract at diagnosis [45]. Next, to model our poverty measure after those that have been most validated, we defined the following neighborhoods: high poverty (30% or more poor), middle poverty (5-29%) and low poverty (less than 5% poor) [10-14,50-52]. The prevalence of poverty in the typical high poverty neighborhood was 36%, just about mid-point between Wilson’s (30%) and Jargowsky and Bane’s (40%) criterion definitions of high poverty. And the typically, quite low, annual household income in such high poverty neighborhoods of $23,150 was much lower than that of typical households in middle ($42,475) or low poverty ($75,050) neighborhoods. Health insurance status, defined as the primary source of payment to the hospital or primary payer, was determined from medical records during the initial course of cancer directed treatment. It was categorized as follows: uninsured (self-pay or no source of payment), Medicaid, Medicare (with or without any supplementation or any other non-means-test-based governmental payer: Veterans Affairs, TRICARE, the health care program for military service members, retirees and their families or the Indian Public Health Service) or private insurer (any managed care corporation or fee-for-service provider).

#### Colon cancer care

Variables were routinely extracted from hospital and physicians’ office patient charts and clinic reports and coded by the California cancer registry [53-55]. These were stage of disease at diagnosis (according to the American Joint Committee on Cancer guidelines) [54], receipt of initial surgery, number of regional lymph nodes harvested for evaluation, receipt of adjuvant chemotherapy, wait times from diagnosis to surgery and chemotherapy, and survival time from diagnosis to death or last follow-up to 10 years. Defining characteristics of cancer stages were stage I (invasion into bowel wall muscle), stage II (invasion through bowel wall muscle), stage III (metastasized to at least 1 regional lymph node) and stage IV (distally metastasized). Stage 0 or in situ tumors were not sampled. Previous research has suggested a range of harvested lymph nodes, from 10 to 16 or more, as well as a range of surgical and post-surgical wait times for chemotherapy (1 month, 2 months or 3 months) that may be of clinical importance [56-62]. They were all examined.

### Statistical analyses

We used logistic or binomial regression models to test hypotheses about the mediating and interaction effects of poverty and payer in predicting binary (survived or not) all-cause colon cancer survival [63-65]. Odds ratios (ORs) and 95% confidence intervals (CIs) were estimated. Modest amounts of missing data would not enter regression models as discrete variables (yes or no) so they probably could not have confounded these analyses. We tested 2-way (gender-by-poverty and gender-by-payer) and 3-way interactions (gender-by-poverty-by-payer) that involved gender. When any of these interactions were significant we assumed the pattern of findings to be different for women and men and reported them separately. Remarkably similar poverty-payer-survival patterns were observed across 2- to 10-year survival analyses. The 6-year analyses were reported as they were the best fit for women and men (Hosmer-Lemeshow test [63,66]).

Logistic regressions were used as statistical tests of complex models involving mediating and moderating effects and their ORs estimate the relative predictive weights of such interacting effects along with main effects. However, under the circumstances of this study, where both “exposures” (e.g., a third of the sample was selected from high poverty neighborhoods) and key outcomes (e.g., 6-year survival was 46% for the entire sample) are common, ORs probably overestimate rate ratios (RRs) [67]. So we provided accompanying practical assessments more germane perhaps to clinical and policy significance. All rates were directly adjusted for relevant covariates (age, stage, poverty and/or payers) using this study’s sample as the standard. Then we used standardized RRs for all between-group comparisons with 95% CIs derived from the Mantel-Haenszel χ^2^ test [68,69]. The statistical interaction is displayed at the bottom of Table 3, Corresponding practical significance indices (RRs) can be found in the Additional file. Finally, this study was reviewed and cleared by the University of Windsor’s research ethics board.

## Results

### Sample description

The two key study variables of neighborhood poverty and primary payer are cross-tabulated in Table 1. People with colon cancer living in high poverty California neighborhoods were nearly twice as likely to be uninsured, five to nearly seven times as likely to be primarily insured by Medicaid, but only two-thirds to half as likely to be so covered by a private insurer as were their counterparts in low poverty neighborhoods. In a practical sense, all of the disadvantages seemed to be substantively greater for women. More detailed descriptive profiles of the California colon cancer patients in our sample are displayed in Table 2. A few characteristics stand out. This sample seems representative of California’s diverse places, including high poverty neighborhoods where 30% or more of the households have aggregate annual incomes below the poverty live. As planned, a third of our sample lived there, while another third resided in so-called middle poverty neighborhoods that actually are still quite poor, with 5% to 29% of the household living below the poverty line. Demographically, our sample of generally older people with colon cancer seems consistent with expectations, nearly half of them covered by Medicare (44%). The relatively low representation of the uninsured (8%) may seem surprising. The vast majority of colon cancer care took place in hospitals where social work staff would work to connect patients who may have been uninsured at the time of their admission to any additional resources such as Medicaid or Medicare for which they qualified by virtue of being poor, older or disabled.

**Table 1:** **Primary payers among women and men with colon cancer diagnosed between 1996 and 2000 in low**, **middle and high poverty neighborhoods**: **prevalence estimates and prevalence ratios**

**Neighborhood poverty**	**Women**				**Men**			
**By primary payer**	**No**.^**a**^	**Prevalence**	**PR**	**(95%CI)**	**No**.^**a**^	**Prevalence**	**PR**	**(95%CI)**
Low Poverty								
Uninsured	60	.056	1.00		67	.066	1.00	
Medicaid	12	.017	1.00		16	.014	1.00	
Medicare	437	.402	1.00		358	.381	1.00	
Private	552	.535	1.00		598	.542	1.00	
Middle Poverty								
Uninsured	74	.069	1.23	(0.91, 1.67)	77	.075	1.14	(0.83, 1.56)
Medicaid	48	.050	**2**.**94**	(1.79, 4.83)	26	.026	**1**.**86**	(1.04, 3.33)
Medicare	549	.453	**1**.**13**	(1.02, 1.25)	408	.436	**1**.**14**	(1.03, 1.26)
Private	438	.430	**0**.**80**	(0.73, 0.87)	480	.467	**0**.**86**	(0.79, 0.94)
High Poverty								
Uninsured	105	.098	**1**.**75**	(1.30, 2.35)	107	.103	**1**.**56**	(1.17, 2.07)
Medicaid	110	.110	**6**.**47**	(4.29, 9.75)	72	.068	**4**.**86**	(2.89, 7.93)
Medicare	587	.501	**1**.**25**	(1.14, 1.37)	451	.486	**1**.**28**	(1.16, 1.42)
Private	319	.295	**0**.**55**	(0.50, 0.61)	349	.347	**0**.**64**	(0.58, 0.71)

Notes. PR = standardized prevalence ratio, CI = confidence interval. Confidence intervals were derived from the Mantel-Haenszel χ^2^ test. All adjustments were internal and direct, using this study’s population of colon cancer cases as the standard. A prevalence ratio of 1.00 was the between-place baseline. Middle and high poverty neighborhood payer prevalence estimates were compared to payer prevalence estimates in low poverty places.

^a^ Number of incident colon cancer cases.

^b^ Prevalence estimates were age-adjusted across these categories: 25–59, 60–69, 70–79 and 80 or older.

**Table 2:** **Stratification**, **demographic**, **payer**, **investigation**, **staging and care characteristics of colon cancer patients diagnosed between 1996 and 2000 and followed until 2011**

	**Sample**	**%**		**Sample**	**%**
Stratification Characteristics					
Places			Poverty prevalence (%) in neighborhood		
Large urban	2,100	33.3	< 5	2,100	33.3
Smaller urban	2,100	33.3	5–29	2,100	33.3
Rural	2,100	33.3	≥ 30	2,100	33.3
Demographic Characteristics					
Age at diagnosis, y			Gender		
25–59	1,347	21.4	Female	3,291	52.2
60–69	1,372	21.8	Male	3,009	47.8
70–79	1,919	30.5			
≥ 80	1,662	26.3			
Race/ethnicity			Primary Payers		
Non-Hispanic white	4,199	66.7	Medicare	2,790	44.3
Non-Hispanic black	688	10.9	Private insurers	2,736	43.4
Hispanic	810	12.9	Medicaid	284	4.5
API or American Indian	603	9.6	Uninsured	490	7.8
Investigation and Staging Characteristics					
Number of regional lymph nodes examined			Stage at diagnosis^a^		
< 6	2,199	37.5	I	1,300	22.5
6–10	1,513	25.8	II	1,882	32.6
11–14	874	14.9	III	1,392	24.1
≥ 15	1,278	21.8	IV	1,202	20.8
Missing data	436	6.9	Missing data	524	8.3
Cancer Care Characteristics					
Received surgery^b^	5,506	88.7	Received chemotherapy	1,751	28.3
Missing data	90	1.4	Missing data	115	1.8
Wait time from diagnosis to surgery, d			Wait time after surgery for chemotherapy, d		
< 14	3,960	71.7	< 14	194	12.0
14–29	1,031	18.7	14–29	415	25.7
30–59	408	7.4	30–59	733	45.3
≥ 60	120	2.2	60–89	159	9.8
Missing data	781	12.4	≥ 90	116	7.2
			Missing data	134	7.6

Note. API = Asian-Pacific Islander.

^a^ American Joint Commission on Cancer staging [54].

^b^ 5,227 were resections and 279 were local excisions.

### Mediation (poverty-payer-survival) and moderation (poverty-by-payer) survival hypotheses

Single predictor and full logistic regression models for 6-year colon cancer survival among women and men are displayed in Table 3. Substantial support for both the mediation and moderation hypotheses was observed for women, but not for men. The top, left column, shows significant main effects of poverty and all payers (versus being uninsured) among women when these factors entered regression models alone. Moving down the column to the full model for women, consistent with mediation, the table shows that in the presence of payer the effect of poverty disappeared and effects of all of the payers were maintained or perhaps even strengthened. The 6-year survival rates among women whose primary payers were Medicaid (RR = 1.83), Medicare (RR = 1.92) or a private insurer (RR = 1.83) were all nearly twice that of uninsured women only 26% of whom so survived.

**Table 3:** **Logistic regression results for main effects and interactions of neighborhood poverty and primary payer by gender on 6**-**year colon cancer survival**

	**Women**					**Men**			
**Predictor variables**	**No**.^**a**^		**OR**		**(95%CI)**	**No**.^**a**^		**OR**	**(95%CI)**
Single predictor models									
Neighborhood poverty									
< 5% poor	1,061		1.00			1,039		1.00	
5-29% poor	1,109		**0.76**		(0.64, 0.91)	991		**0**.**68**	(0.57, 0.82)
≥ 30% poor	1,121		**0.62**		(0.52, 0.74)	979		**0**.**57**	(0.48, 0.69)
Primary payer									
Uninsured	239		1.00			251		1.00	
Medicaid	170		**2.01**		(1.31, 3.08)	114		1.24	(0.78, 1.99)
Medicare	1,573		**2.48**		(1.79, 3.43)	1,217		**1**.**82**	(1.34, 2.48)
Private	1,309		**2.78**		(2.02, 3.83)	1,427		**2**.**36**	(1.76, 3.16)
Full models									
Neighborhood poverty									
< 5% poor	1,061		1.00			1,039		1.00	
5-29% poor	1,109		1.07		(0.78, 1.48)	991		**0**.**70**	(0.58, 0.84)
≥ 30% poor	1,121		1.25		(0.71, 2.18)	979		**0**.**62**	(0.52, 0.75)
Primary payer									
Uninsured	239		1.00			251		1.00	
Medicaid	170		**2**.**40**		(1.55, 3.73)	114		1.33	(0.83, 2.12)
Medicare	1,573		**3.93**		(2.33, 6.64)	1,217		**1**.**79**	(1.31, 2.44)
Private	1,309		**3**.**66**		(2.36, 5.67)	1,427		**2**.**19**	(1.63, 2.93)
Poverty by payer	3,291		**0**.**87**		(0.78, 0.98)	Null interaction removed			
Poverty by payer interaction among women									
	> 30% poor			5-29% poor			< 5% poor		
	No.^a^	OR	(95% CI)	No.^a^	OR	(95% CI)	No.^a^	OR	(95% CI)
Primary payer									
Uninsured	105	1.00		74	1.00		60	1.00	
Medicaid	110	1.60	(0.90, 2.84)	48	1.83	(0.82, 4.07)	12	**5**.**94**	(1.50, 23.54)
Medicare	587	**1**.**68**	(1.03, 2.74)	549	**2**.**65**	(1.48, 4.75)	437	**4**.**69**	(2.30, 9.57)
Private	319	**1**.**92**	(1.18, 3.13)	438	**2**.**61**	(1.47, 4.64)	552	**5**.**03**	(2.51, 10.08)

Notes. OR = odds ratio, CI = confidence interval. All effects were age-adjusted across these categories: 25–59, 60–69, 70–79 and 80 or older. After age, poverty and payer were accounted for, place (large urban, smaller urban, rural) did not enter the full models for women or men.

^a^ Number of incident colon cancer cases.

The hypothesis that the payer-colon cancer survival relationship would be moderated by poverty was also supported for women, but not for men. The statistical interaction is displayed at the bottom of Table 3, while the practical effect moderation is depicted at the bottom of Additional file 1. Among women, all of the payers seemed less effective in high poverty neighborhoods than in low poverty neighborhoods and Medicaid did not seem any more effective than having no insurance. Consistent with the theory that more facilitative social and economic capital is available in more affluent, low poverty neighborhoods, the 6-year survival rates there among women whose primary payers were Medicaid (RR = 2.90), Medicare (RR = 2.91) or a private insurer (RR = 2.60) were all nearly three times that of uninsured women who lived there. As for men, only significant main effects of poverty and payer were observed.

### Effects of being uninsured or underinsured and living in extremely poor neighborhoods on colon cancer investigations, diagnoses and treatments

#### Investigation and stage at diagnosis

Generally modest effects of poverty and payer that did not differ significantly by gender demonstrated the lymph node investigation criterion of 15 or more to be most predictive in this sample. A significant poverty by payer interaction was also found (left column of Table 4). In low poverty areas, colon cancer patients whose primary payer was Medicare were nearly twice as likely (RR = 1.91) to have had 15 or more regional lymph nodes harvested during surgery for diagnostic evaluation than were the uninsured. Trends in the same direction that were not statistically significant, however, were observed for those primarily covered by Medicaid or private insurers. The combined any payer versus uninsured effect among those who lived in such more affluent neighborhoods approached significance (RR = 1.64; 90% CI = 1.06, 2.53, data not shown). No such effects of any payers were observed in high or middle poverty neighborhoods. There were only, relatively small, main effects of poverty and payer on early diagnosis of stage I or stage II disease.

**Table 4:** Logistic regression results for main effects and interactions of neighborhood poverty and primary payer on high quality investigation and early stage at diagnosis

	**15 or more regional lymph nodes examined**					**Stage I or stage II at the time of diagnosis**			
**Predictor variables**	**No**.^**a**^		**OR**		**(95%CI)**	**No**.^**a**^		**OR**	**(95%CI)**
Single predictor models									
Neighborhood poverty									
< 5% poor	1,727		1.00			1,959		1.00	
5-29% poor	1,646		0.92		(0.79, 1.07)	1,923		**0**.**81**	(0.72, 0.92)
≥ 30% poor	1,583		0.95		(0.81, 1.11)	1,894		0.89^*^	(0.79, 1.01)
Primary payer									
Uninsured	241		1.00			310		1.00	
Medicaid	229		2.01		(0.74, 1.65)	261		1.06	(0.77, 1.48)
Medicare	2,221		1.11		(0.81, 1.53)	2,601		**1**.**33**	(1.04, 1.71)
Private	2,265		0.97		(0.72, 1.30)	2,604		**1**.**30**	(1.03, 1.65)
Full models									
Neighborhood poverty									
< 5% poor	1,727		1.00			1,959		1.00	
5-29% poor	1,646		1.34^*^		(0.99, 1.80)	1,923		**0**.**82**	(0.72, 0.93)
≥ 30% poor	1,583		**1**.**97**		(1.17, 3.32)	1,894		0.91	(0.80, 1.04)
Primary payer									
Uninsured	241		1.00			310		1.00	
Medicaid	229		1.30		(0.86, 1.98)	261		1.07	(0.77, 1.49)
Medicare	2,221		**2**.**03**		(1.22, 3.41)	2,601		**1**.**33**	(1.03, 1.70)
Private	2,265		1.49^*^		(0.97, 2.29)	2,604		**1**.**28**	(1.01, 1.63)
Poverty by payer	4,956		**0**.**85**		(0.76, 0.94)	Null interaction removed			
Poverty by payer interaction on high quality investigation									
	> 30% poor			5-29% poor			< 5% poor		
	No.^a^	OR	(95% CI)	No.^a^	OR	(95% CI)	No.^a^	OR	(95% CI)
Primary payer									
Uninsured	114	1.00		72	1.00		55	1.00	
Medicaid	144	1.11	(0.65, 1.88)	63	0.70	(0.32, 1.53)	22	1.91	(0.63, 5.79)
Medicare	794	0.93	(0.58, 1.48)	753	0.92	(0.52, 1.63)	674	**2**.**01**	(1.02, 3.99)
Private	531	1.00	(0.64, 1.57)	758	0.85	(0.50, 1.45)	976	1.32	(0.68, 2.55)

Notes. OR = odds ratio, CI = confidence interval. All effects were age-adjusted across these categories: 25–59, 60–69, 70–79, and 80 or older.

^a^ Number of incident colon cancer cases.

^*^ p < .10.

#### Surgery and adjuvant chemotherapy

The effects of poverty and payer on the receipt of surgery for colon cancer patients with non-metastasized disease are displayed in Table 5. There were no apparent interaction effects. Full statistical models suggested disadvantaging effects of living in high poverty neighborhoods among women and middle poverty neighborhoods among men. They also strongly suggested the protective effects of Medicaid coverage for women (OR = 10.14) and private health insurance coverage for men (OR = 9.98). In a practical sense, any health insurance coverage (private, Medicare or Medicaid) increased surgical rates by 13% to 16% (RRs of 1.13 to 1.16). While practically all insured patients received such indicated care, only 86% of uninsured patients did. There were no significant main or interaction effects of poverty or primary payer on any surgical wait times.

**Table 5:** **Logistic regression results for main effects of neighborhood poverty and primary payer by gender on receipt of colon cancer surgery among patients with non**-**metastasized disease**

	**Women**			**Men**		
**Predictor variables**	**No**.^**a**^	**OR**	**(95%CI)**	**No**.^**a**^	**OR**	**(95%CI)**
Single predictor models						
Neighborhood poverty						
< 5% poor	774	1.00		713	1.00	
5-29% poor	762	0.50^*^	(0.24, 1.06)	675	**0**.**32**	(0.11, 0.90)
≥ 30% poor	761	**0**.**38**	(0.19, 0.78)	637	0.35^*^	(0.12, 1.01)
Primary payer						
Uninsured	94	1.00		109	1.00	
Medicaid	113	**9**.**66**	(1.12, 83.68)	74	2.49	(0.26, 23.47)
Medicare	1,135	**5**.**12**	(1.85, 14.20)	844	** 3**.**80**	(1.06, 13.58)
Private	955	**3**.**39**	(1.30, 8.88)	998	**10**.**09**	(2.62, 38.85)
Full models						
< 5% poor	774	1.00	7.13	1.00		
5-29% poor	762	0.49^*^	(0.23, 1.03)	675	**0**.**32**	(0.11, 0.91)
≥ 30% poor	761	**0**.**37**	(0.18, 0.77)	637	0.41	(0.14, 1.19)
Primary payer						
Uninsured	94	1.00		109	1.00	
Medicaid	113	**10**.**14**	(1.17, 87.92)	74	2.82	(0.30, 26.85)
Medicare	1,135	** 4**.**43**	(1.58, 12.47)	844	**3**.**93**	(1.08, 14.27)
Private	955	2.65^*^	(0.99, 7.08)	998	**9**.**98**	(2.53, 39.33)

Notes. OR = odds ratio, CI = confidence interval. All effects were age- and stage-adjusted across these categories: 25–59, 60–69, 70–79 and 80 or older; and stage I, stage II and stage III.

* p < .10.

The effects of poverty and payer on the receipt of adjuvant chemotherapy for colon cancer patients with stage II or stage III disease are displayed in Table 6. Three findings stand out. First, patients who lived in high poverty neighborhoods were 18% less likely to have received post-surgical chemotherapy than were their counterparts in low poverty neighborhoods (OR = 0.65, RR = 0.82). Second, the statistical models suggested that the poverty-chemotherapy wait time relationship might be mediated by payer as there seemed some attenuation of that poverty-therapy relationship in the presence of payer (unadjusted OR = 1.55; 95% CI = 1.04, 2.30 and adjusted OR = 1.33; 95% CI = 0.88, 2.02). In practical terms though, poverty and payer seemed independently important. The very poor were much more likely than the affluent to wait for 2 months or more after surgery to receive chemotherapy (RR = 1.38) and those with Medicare or private health insurance were half as likely as the uninsured or those with Medicaid to wait that long (RR = 0.52).

**Table 6:** **Logistic regression results for main effects of neighborhood poverty and primary payer on chemotherapy receipt and post**-**surgical wait for chemotherapy of 60 days or more among patients with stage II or stage III colon cancer**

	**Single predictor models**			**Full models**		
**Predictor variables**	**No**.^**a**^	**OR**	**(95%CI)**	**No**.^**a**^	**OR**	**(95%CI)**
Received chemotherapy after surgery						
Neighborhood poverty						
< 5% poor	1,103	1.00		1,103	1.00	
5-29% poor	1,066	**0**.**80**	(0.65, 0.98)	1,066	**0**.**80**	(0.65, 0.98)
≥ 30% poor	1,045	**0**.**63**	(0.52, 0.78)	1,045	**0**.**65**	(0.52, 0.80)
Primary payer						
Uninsured	164	1.00		164	1.00	
Medicaid	147	**0**.**49**	(0.30, 0.81)	147	**0**.**51**	(0.31, 0.85)
Medicare	1,436	0.75	(0.51, 1.11)	1,436	0.71^*^	(0.48, 1.04)
Private	1,467	0.85	(0.60, 1.22)	1,467	0.75	(0.52, 1.09)
Waited 60 days or more for adjuvant chemotherapy						
Neighborhood poverty						
< 5% poor	421	1.00		421	1.00	
5-29% poor	369	**1**.**48**	(1.01, 2.18)	369	1.45^*^	(0.98, 2.14)
> 30% poor	326	**1**.**55**	(1.04, 2.30)	326	1.33	(0.88, 2.02)
Primary payer						
Uninsured	90	1.00		90	1.00	
Medicaid	58	0.87	(0.40, 1.86)	58	0.85	(0.39, 1.83)
Medicare	364	**0**.**42**	(0.24, 0.76)	364	**0**.**43**	(0.24, 0.77)
Private	604	**0**.**41**	(0.24, 0.69)	604	**0**.**43**	(0.25, 0.73)

Notes. OR = odds ratio, CI = confidence interval. All effects were age- and stage-adjusted across these categories: 25–59, 60–69, 70–79 and 80 or older; and stage II or stage III.

* p < .10.

We developed a nominal measure of optimum treatment of stage III colon cancer from four study variables: received colon-cancer directed surgery within 30 days of diagnosis and received adjuvant chemotherapy within 45 days of surgery. Admittedly, it is probably only one of a number of “optimum” care criterions with some measure of clinical validity. A statistical main effect of poverty was observed for women and a poverty-by-payer interaction effect was observed for men (Table 7). In low poverty neighborhoods about 7 of every 10 patients with stage III colon cancer received such optimum care and this outcome did not differ significantly between women and men. In high poverty neighborhoods, however, 20% fewer women received such optimum care (RR = 0.80). For men, there was no main effect of poverty, but there was an effect of payer in high poverty neighborhoods. Only 4 of every 10 such men in very poor neighborhoods who were either uninsured or insured by Medicaid received optimum care. Similarly poor men with Medicare or private health insurance were more than 60% more likely to have received optimum care (RR = 1.62). We also compared very poor uninsured men with very poor men who were insured by any payer, public or private. The vast majority of such uninsured men did not receive optimum care (29%). However, very poor men with any type of health insurance were more than twice as likely to have received optimum care (RR = 2.30; 95% CI = 1.10, 1.43, data not shown). Rates for patient refusal of chemotherapy were similar (3% overall) between poverty and payer groups.

**Table 7:** **logistic regression results for main effects and interactions of neighborhood poverty and primary payer by gender on receipt of optimum treatment**^**a**^**among colon cancer patients with stage III disease**

	**Women**			**Men**		
**Predictor variables**	**No**.^**b**^	**OR**	**(95%CI)**	**No**.^**b**^	**OR**	**(95%CI)**
Single predictor models						
Neighborhood poverty						
< 5% poor	133	1.00		133	1.00	
5-29% poor	131	**0**.**53**	(0.32, 0.88)	115	0.98	(0.58, 1.66)
≥ 30% poor	109	**0**.**55**	(0.32, 0.95)	103	0.88	(0.51, 1.50)
Primary payer						
Uninsured	29	1.00		27	1.00	
Medicaid	24	0.84	(0.26, 2.72)	15	0.67	(0.19, 2.42)
Medicare	131	0.52	(0.21, 1.30)	119	1.47	(0.59, 3.66)
Private	189	0.86	(0.37, 2.00)	190	1.06	(0.46, 2.44)
Full models						
Neighborhood poverty						
< 5% poor	133	1.00		133	1.00	
5-29% poor	131	**0**.**53**	(0.32, 0.89)	115	**0**.**28**	(0.10, 0.77)
≥ 30% poor	109	**0**.**55**	(0.31, 0.98)	103	**0**.**08**	(0.01, 0.47)
Primary payer						
Uninsured	29	1.00		27	1.00	
Medicaid	24	0.82	(0.25, 2.68)	15	0.41	(0.10, 1.70)
Medicare	131	0.47	(0.19, 1.21)	119	0.22^*^	(0.04, 1.20)
Private	189	0.72	(0.30, 1.72)	190	0.27^*^	(0.07, 1.06)
Poverty by payer	Null Interaction removed			351	**1**.**75**	(1.19, 2.57)
Poverty by payer interaction among men						
	> 30% poor			< 30% poor		
	No.^b^	OR	(95% CI)	No.^b^	OR	(95% CI)
Primary payer						
Uninsured	10	1.00		17	1.00	
Medicaid	9	3.03	(0.43, 21.33)	6	0.22	(0.03, 1.69)
Medicare	37	**13**.**33**	(2.42, 73.50)	82	0.40	(0.10, 1.60)
Private	47	3.69^*^	(0.81, 16.84)	143	0.41	(0.11, 1.51)

Notes. OR = odds ratio, CI = confidence interval. All effects were age-adjusted across these categories: 25–59, 60–69, 70–79 and 80 or older.

^a^ Optimum treatment: received colon cancer-directed surgery within 30 days of diagnosis and received adjuvant chemotherapy within 45 days of surgery.

^b^ Number of incident colon cancer cases.

* p < .10.

#### Experimental and palliative chemotherapies

Chemotherapy for stage II colon cancer, experimental at the time of this study was not associated with gender or payer. But it was less likely to have been received by patients in mid- to high poverty (17%) than in low poverty (25%) neighborhoods (RR = 0.68; 95% CI = 0.57, 0.82). We also found a poverty-by-payer interaction such that in middle poverty neighborhoods the privately insured may have been slightly more likely to have received chemotherapy than were others, respectively, 24% and 18% (RR = 1.29; 90% CI = 1.00, 1.66). Finally, we considered receipt of palliative chemotherapy by those with stage IV colon cancer. Straightforward independent main disadvantaging effects of mid- to high poverty (44% versus 56%, RR = 0.80; 95% CI = 0.71, 0.90) and being uninsured or covered by Medicaid (38% versus 51%, RR = 0.75; 95% CI = 0.62, 0.91) that did not differ between women and men were observed.

## Discussion

To the best of our knowledge, this is the first report of the combined effects of poverty and health insurance on colon cancer care in high poverty US neighborhoods. We found strong support for our hypothesis that health insurance mediates the poverty-colon cancer survival relationship for women. The apparent effect of poverty disappeared in the presence of payer and the effects of all forms of health insurance seemed strengthened. All were associated with substantial survival advantages compared to the uninsured: Medicaid (RR = 1.83), Medicare (RR = 1.92) and private (RR = 1.83). We also found support for our poverty-by-health insurance interaction hypothesis for women. The survival effects of all forms of health insurance were stronger in low poverty neighborhoods than they were in poor to very poor neighborhoods: Medicaid (RR = 2.90), Medicare (RR = 2.91) and private (RR = 2.60). It seems that the effectiveness of all health insurance programs is significantly impacted by the availability of other key resources. In more well to do neighborhoods where social and economic capital abounds most people with colon cancer seem quite able to absorb the indirect and additional uncovered, direct costs of cancer care. High poverty neighborhoods on the other hand, with their relative lack of such capital reserves, seem to remain as described more than a generation ago by William Julius Wilson, places of “true disadvantage” [10], places of multiplicative disadvantage, especially for the women who live there. Not only are they much more likely to be uninsured (2 times as likely as less poor women) or underinsured (more than 6 times as likely to be covered by Medicaid), but when insured, all such insurance programs, public and private, seem to be much less effective there than they are in less poor places. There is even some evidence to suggest that being covered by Medicaid may be no more beneficial than being uninsured among many women who live in such extremely poor places.

For men, poverty and health insurance still seem to matter very much, but in more straightforward ways, as independent main effects. Across colon cancer care processes, poverty and public payers, particularly Medicare, seemed more instrumental for women while private payers slightly more so for men. Such stands to reason given the facts that other than government health care insurance programs are predominantly purchased through employers in the US and over the life times of these generally now older people with colon cancer such opportunities have been much more available to men than to women.

### Potential limitations

#### Internal validity

Our use of ecological poverty measures might suggest alternative explanations for our results though we intended them to be contextual neighborhood measures, rather than compositional personal measures. Still, one might wonder if the racial/ethnic composition of high poverty neighborhoods, rather than their concentration of poor households, accounted for the colon cancer survival differences we observed. First, recent US studies of colon cancer diagnosis, treatment and survival have consistently found that socioeconomic differences explained most racial-group differences [26,70-74]. Second, although we did not account for this factor in the present analysis as it was outside its scope, we are concomitantly performing related analyses that will soon be reported. We have found, for example, that health insurance also mediates ethnicity-breast cancer care relationships, accounting for many of the disadvantages observed among Hispanic women. In short, while not refuting previous inferences about the importance of race, ethnicity and culture in cancer care, our analyses suggest that having adequate health insurance is probably much more important.

For now, we do not think that race/ethnicity confounded this analysis for the following reasons. Two-thirds of our sample was comprised of non-Hispanic white people. Even within our subsample of high poverty neighborhood residents, nearly half of them were non-Hispanic white people. Most importantly, we were able to systematically replicate both our mediation and moderation hypothesis tests for non-Hispanic white women and women of color (Hispanic, non-Hispanic black, Asian-Pacific Islanders and American Indian people) as well as systematically replicate similar main effects of poverty and health insurance for non-Hispanic men and for men of color.

There has been a largely unmet need for research on the construct validity of ecological socioeconomic measures that are so often used in public health research. Wilson, Jargowsky and Bane added much relevant knowledge on high poverty neighborhood measures [10-12], and our research group has done some work to advance understandings of poor neighborhoods, analogous to this study’s middle poverty neighborhoods [2,3]. They seem prevalently represented by not only the poor, but the near poor and working poor as well as lower-middle and middle class people. This study added further to our knowledge about vulnerable neighborhoods. For the first time that we are aware, steep monotonic gradients were observed for various types of health insurance across low poverty (e.g., baseline Medicaid coverage of women), middle poverty (prevalence 3-fold greater) and high poverty neighborhoods (prevalence more than 6-fold greater, Table 1[75,76]). This new knowledge concerning the construct validity of ecological poverty measures may advance our understanding about the context in which very poor Americans live. Therefore, our findings are probably not prone to ecological fallacy. They may, in fact, help researchers to better understand their contextual measures and so avoid individualistic fallacies of inference [77,78]. Finally, the preferential predictive validity of our census tract-based poverty measure was consistent with that observed by others [50,51]. After such poverty measures entered this study’s analytic models, no education or occupation-based measures did.

Another possible limitation of our study was incomplete information on outpatient treatments [79,80]. Such data are more difficult for cancer registries to collect than inpatient data. However, the California cancer registry has been shown to be nearly complete for chemotherapy data (85%) in one place that we sampled, San Francisco [81]. In addition, analyses of hospital-based surgery, lymph node evaluation, stage and survival were unlikely to have been affected [82], and missing chemotherapy data were not prevalent in our sample.

We focused on all-cause or overall survival, rather than cancer-specific survival. Cancer was the underlying cause of death among the vast majority of patients in our sample (72%) who died during the study period, accounting for nearly 8 of every 10 deaths (78%), for example, among perhaps the most policy-interesting group for its observed socioeconomic gradients, those with stage III colon cancer. Although vital status and length of overall survival are highly accurate in cancer registries, the underlying cause of death probably is not [83-85]. Although death certificate error was a likely limitation, we did systematically replicate our central all-cause survival hypothesis tests with cancer-specific ones. If anything, our overall survival effects were probably slight underestimates of cancer-specific ones [84]. Finally, the underlying cause of many deaths not coded as cancer mortality can be directly associated with lack of treatment or with cancer treatment complications [86]. For all of these reasons, we think overall survival a more policy-telling indicator of practical clinical and human significance.

#### External validity

This study’s sample of people with colon cancer may not be generalizable to all such people across the US. Purposively diverse and potentially policy-important places in California were oversampled: high poverty neighborhoods in very large urban to rural places. Admittedly, our findings are most generalizable to such places. Previous research has suggested that such high poverty places probably have some important commonalities across America [10-12]. It should also be noted that after accounting for demographic, socioeconomic and clinical factors, place (large urban, smaller urban or rural) did not seem to matter in this study’s analytic models. This study’s inferences, therefore, can probably be confidently generalized across many states, especially to their high poverty neighborhoods. Finally, one may wonder about the apparent non-significance of geographic place in these analyses. Are there not critical elements of geography that are already well known to be associated with health care access and effectiveness: urbanity, health care service endowments, distances to such services and the like? Surely there are and so, yes, geographic place matters. Our analysis points out though that the socioeconomic elements of place, particularly the main, mediating and moderating effects of highly concentrated impoverishment, probably matters much more.

We think that some of our analyses were statistically powerful, especially, tests of poverty-health insurance-colon cancer survival mediation hypotheses that typically analyzed survival among approximately 3,000 women and 3,000 men with approximately 1,000 study participants in each of three poverty groups. Even their uninsured subsamples in the range of 250 to 300 probably ensured detection of important effects. These analyses tended to provide rather precise effect estimates, engendering substantial scientific and policy confidence. Admittedly, a few of the moderator hypotheses we examined, especially those related to the increasingly specific experiences, for example, of uninsured or underinsured women or men in high poverty neighborhoods who were treated optimally for stage III colon cancer, where key subsamples ranged as low as 10 to 25, were necessarily exploratory. We hope that other researchers, especially those with access to national cancer data, will systematically replicate and extend our analyses.

## Conclusions

Health insurance substantially mediates the quality of colon cancer care in the United States. Moreover, poverty seems to make the effects of being uninsured or underinsured even worse, especially among women. These findings are consistent with the theory that more facilitative social and economic capital is available in more affluent neighborhoods, where people with cancer may be better able to absorb the indirect and uncovered direct costs of care. Policy makers need to understand that even covered health care presently comes with myriad such costs. And though some are able to absorb them, many others are not.

## Competing interests

The authors declare that they have no competing interests.

## Authors’ contributions

KMG conceptualized and supervised the study and led the writing. KMG, INL, EJH, GYZ and CH obtained funding. GYZ supervised the analysis. All authors assisted with study design, data analysis and interpretation, and writing. All authors read and approved the final manuscript.

## Pre-publication history

The pre-publication history for this paper can be accessed here:

http://www.biomedcentral.com/1471-2458/12/897/prepub

## Supplementary Material

Addtional file 1 Additional tabular presentations related to practical significance: Rates and rate ratios. Click here for file
