# Supplementary material for: Effects of being uninsured or underinsured and living in extremely poor neighborhoods on colon cancer care and survival in California: historical cohort analysis, 1996—2011
Source: BMC Public Health. 2012 Oct 24;12:897. doi: 10.1186/1471-2458-12-897 (PMC3507906; doi:10.1186/1471-2458-12-897)
Supplement: Addtional file 1 — Additional tabular presentations related to practical significance: Rates and rate ratios. [file 1471-2458-12-897-S1.docx]

**SUPPLEMENTAL TABLES**

**TABLE 3S—Main Effects and Interactions of Neighborhood Poverty and Primary Payer by Gender on 6-Year Colon Cancer Survival: Survival Rates and Survival Rate Ratios**

______________________________________________________________________________

Women Men

No.^a^ Rate RR (95% CI) No.^a^ Rate RR (95% CI)

Independent main effects

Neighborhood poverty^b^

< 5% poor 1,061 .509 1.00 1,039 .496 1.00

5-29% poor 1,109 .443 **0.87** (0.80, 0.95) 991 .404 **0.81** (0.77, 0.85)

> 30% poor 1,121 .348 **0.68** (0.62, 0.75) 979 .367 **0.74** (0.70, 0.78)

Primary payer^b^

Uninsured 239 .272 1.00 251 .256 1.00

Medicaid 170 .435 **1.60** (1.40, 1.83) 114 .419 **1.64** (1.41, 1.91)

Medicare 1,573 .476 **1.75** (1.59, 1.92) 1,217 .394 **1.54** (1.46, 1.62)

Private 1,309 .484 **1.78** (1.62, 1.95) 1,427 .465 **1.82** (1.65, 2.00)

Adjusted main effects

Neighborhood poverty^c^

< 5% poor 1,061 .499 1.00 1,039 .493 1.00

5-29% poor 1,109 .441 **0.88** (0.80, 0.97) 991 .400 **0.81** (0.74, 0.89)

> 30% poor 1,121 .404 **0.81** (0.74, 0.89) 979 .396 **0.80** (0.72, 0.88)

Primary payer^d^

Uninsured 239 .259 1.00 251 .256 1.00

Medicaid 170 .473 **1.83** (1.40, 2.39) 114 .422 **1.65** (1.16, 2.34)

Medicare 1,573 .498 **1.92** (1.60, 2.30) 1,217 .392 **1.53** (1.25, 1.88)

Private 1,309 .475 **1.83** (1.51, 2.22) 1,427 .461 **1.80** (1.49, 2.17)

______________________________________________________________________________

Poverty by payer interaction among women

> 30% poor 5-29% poor < 5% poor

No.^a^ Rate RR (95% CI) No.^a^ Rate RR (95% CI) No.^a^ Rate RR (95% CI)

______________________________________________________________________________

Primary payer^b^

Uninsured 105 .306 1.00 74 .271 1.00 60 .206 1.00

Medicaid 110 .344 1.12 (0.79, 1.58) 48 .483 **1.78** (1.10, 2.87) 12 .597 **2.90** (1.35, 6.24)

Medicare 587 .409 **1.34** (1.01, 1.78) 549 .486 **1.79** (1.29, 2.72) 437 .600 **2.91** (2.02, 4.18)

Private 319 .436 **1.42** (1.06, 1.90) 438 .458 **1.69** (1.20, 2.38) 552 .535 **2.60** (1.77, 3.81)

Notes. RR = standardized rate ratio, CI = confidence interval. Confidence intervals were derived from the Mantel-Haenszel χ^2^ test. All adjustments were internal and direct, using this study’s population of colon cancer cases as the standard.

^a^ Number of incident colon cancer cases.

^b^ Rates were age-adjusted across these categories: 25-59, 60-69, 70-79 and 80 or older.

^c^ Rates were adjusted for age and primary payer across these categories: 25-59, 60-69, 70-79 and 80 or older; and uninsured, Medicaid, Medicare and private insurance.

^d^ Rates were adjusted for age and neighborhood poverty across these categories: 25-59, 60-69, 70-79 and 80 or older; and < 5% poor, 5-29% poor and > 30% poor.

**TABLE 4S—Main Effects and Interactions of Neighborhood Poverty and Primary Payer on High Quality Investigation and Early Stage at Diagnosis: Rates and Rate Ratios**

______________________________________________________________________________

15 or more regional Stage I or stage II

lymph nodes examined at the time of diagnosis

No.^a^ Rate RR (95% CI) No.^a^ Rate RR (95% CI)

Independent main effects

Neighborhood poverty^b^

< 5% poor 1,727 .265 1.00 1,959 .579 1.00

5-29% poor 1,646 .249 0.94 (0.83, 1.05) 1,923 .530 **0.91** (0.86, 0.97)

> 30% poor 1,583 .253 0.95 (0.84, 1.07) 1,894 .552 0.95^*^ (0.90, 1.01)

Primary payer^b^

Uninsured 241 .224 1.00 310 .497 1.00

Medicaid 229 .271 1.21 (0.88, 1.66) 261 .525 1.06 (0.89, 1.26)

Medicare 2,221 .265 1.18 (0.93, 1.49) 2,601 .557 **1.12** (1.00, 1.25)

Private 2,265 .244 1.09 (0.78, 1.39) 2,604 .556 **1.12** (1.00, 2.25)

Adjusted main effects

Neighborhood poverty^c^

< 5% poor 1,727 .267 1.00 1,959 .570 1.00

5-29% poor 1,646 .244 0.91 (0.80, 1.03) 1,923 .524 **0.92** (0.87, 0.97)

> 30% poor 1,583 .271 1.01 (0.94, 1.09) 1,894 .552 0.97 (0.92, 1.02)

Primary payer^d^

Uninsured 241 .208 1.00 310 .496 1.00

Medicaid 229 .252 1.21 (0.88, 1.66) 261 .530 1.07 (0.90, 1.27)

Medicare 2,221 .275 **1.32** (1.04, 1.68) 2,601 .550 1.11^*^ (0.99, 1.24)

Private 2,265 .244 1.17 (0.92, 1.49) 2,604 .554 **1.12** (1.00, 1.26)

______________________________________________________________________________

Poverty by payer interaction on high quality investigation

> 30% poor 5-29% poor < 5% poor

No.^a^ Rate RR (95% CI) No.^a^ Rate RR (95% CI) No.^a^ Rate RR (95% CI)

______________________________________________________________________________

Primary payer^b^

Uninsured 114 .269 1.00 72 .246 1.00 55 .182 1.00

Medicaid 144 .286 1.06 (0.79, 1.42) 63 .183 0.74 (0.36, 1.51) 22 .281 1.54 (0.58, 4.07)

Medicare 794 .243 0.90 (0.58, 1.55) 753 .228 0.93 (0.66, 1.31) 674 .348 **1.91** (1.15, 3.16)

Private 531 .254 0.94 (0.51, 1.72) 758 .239 0.97 (0.72, 1.31) 976 .265 1.46 (0.87, 2.44)

______________________________________________________________________________

Notes. RR = standardized rate ratio, CI = confidence interval. Confidence intervals were derived from the Mantel-Haenszel χ^2^ test. All adjustments were internal and direct, using this study’s population of colon cancer cases as the standard.

^a^ Number of incident colon cancer cases.

^b^ Rates were age-adjusted across these categories: 25-59, 60-69, 70-79 and 80 or older.

^c^ Rates were adjusted for age and primary payer across these categories: 25-59, 60-69, 70-79 and 80 or older; and uninsured, Medicaid, Medicare and private insurance.

^d^ Rates were adjusted for age and neighborhood poverty across these categories: 25-59, 60-69, 70-79 and 80 or older; and < 5% poor, 5-29% poor and > 30% poor.

* p < .10.

**TABLE 5S—Main Effects of Neighborhood Poverty and Primary Payer by Gender on Receipt of Colon Cancer Surgery among Patients with Non-Metastasized Disease: Rates and Rate Ratios**

______________________________________________________________________________

Women Men

No.^a^ Rate RR (95% CI) No.^a^ Rate RR (95% CI)

Independent main effects

Neighborhood poverty^b^

< 5% poor 774 .989 1.00 713 .993 1.00

5-29% poor 762 .978 0.99^*^ (0.98, 1.00) 675 .981 **0.99** (0.98, 1.00)

> 30% poor 761 .968 **0.98** (0.97, 0.99) 637 .983 0.99^*^ (0.98, 1.00)

Primary payer^b^

Uninsured 94 .903 1.00 109 .931 1.00

Medicaid 113 .989 **1.10** (1.03, 1.17) 74 .997 **1.07** (1.01, 1.13)

Medicare 1,135 .986 **1.09** (1.06, 1.12) 844 .983 **1.06** (1.03, 1.09)

Private 955 .978 **1.08** (1.04, 1.12) 998 .995 **1.07** (1.05, 1.09)

Adjusted main effects

Neighborhood poverty^c^

< 5% poor 774 .984 1.00 713 .990 1.00

5-29% poor 762 .976 0.99 (0.97, 1.01) 675 .962 **0.97** (0.95, 0.99)

> 30% poor 761 .960 **0.97** (0.95, 0.99) 637 .982 0.99 (0.97, 1.01)

Primary payer^d^

Uninsured 94 .863 1.00 109 .859 1.00

Medicaid 113 .999 **1.16** (1.08, 1.25) 74 .999 **1.16** (1.06, 1.27)

Medicare 1,135 .984 **1.14** (1.10, 1.18) 844 .981 **1.14** (1.10, 1.19)

Private 955 .971 **1.13** (1.08, 1.18) 998 .993 **1.16** (1.12, 1.20)

______________________________________________________________________________

Notes. RR = standardized rate ratio, CI = confidence interval. Confidence intervals were derived from the Mantel-Haenszel χ^2^ test. All adjustments were internal and direct, using this study’s population of colon cancer cases as the standard.

^a^ Number of incident colon cancer cases.

^b^ Rates were age-adjusted across these categories: 25-59, 60-69, 70-79 and 80 or older.

^c^ Rates were adjusted for age and primary payer across these categories: 25-59, 60-69, 70-79 and 80 or older; and uninsured, Medicaid, Medicare and private insurance.

^d^ Rates were adjusted for age and neighborhood poverty across these categories: 25-59, 60-69, 70-79 and 80 or older; and < 5% poor, 5-29% poor and > 30% poor.

* p < .10.

**TABLE 6S—Main Effects of Neighborhood Poverty and Primary Payer on Chemotherapy Receipt and Post-Surgical Wait for Chemotherapy of 60 Days or More Among Patients with Stage II or Stage III Colon Cancer: Rates and Rate Ratios**

______________________________________________________________________________

Independent effects^a^ Adjusted effects^a^

No.^b^ Rate RR (95% CI) No.^b^ Rate RR (95% CI)

Received chemotherapy after surgery

Neighborhood poverty

< 5% poor 1,103 .377 1.00 1,103 .381^c^ 1.00

5-29% poor 1,066 .352 0.93 (0.83, 1.04) 1,066 .351^c^ 0.92 (0.82, 1.03)

> 30% poor 1,045 .309 **0.82** (0.73, 0.92) 1,045 .313^c^ **0.82** (0.73, 0.92)

Primary payer

Uninsured/Medicaid 311 .373 1.00 311 .380^d^ 1.00

Medicare/Private 2,903 .369 0.99 (0.94, 1.05) 2,903 .366^d^ 0.96 (0.81, 1.14)

Waited 60 days or more for adjuvant chemotherapy

Neighborhood poverty

< 5% poor 421 .167 1.00 421 .169^c^ 1.00

5-29% poor 369 .192 1.15 (0.86, 1.54) 369 .197^c^ 1.17 (0.87, 1.57)

> 30% poor 326 .225 **1.35** (1.00, 1.82) 326 .234^c^ **1.38** (1.03, 1.84)

Primary payer

Uninsured/Medicaid 148 .351 1.00 148 .343^d^ 1.00

Medicare/Private 968 .180 **0.51** (0.39, 0.67) 968 .180^d^ **0.52** (0.40, 0.62)

______________________________________________________________________________

Notes. RR = standardized rate ratio, CI = confidence interval. Confidence intervals were derived from the Mantel-Haenszel χ^2^ test. All adjustments were internal and direct, using this study’s population of colon cancer cases as the standard.

^a^ Rates were age-adjusted across these categories: 25-59, 60-69, 70-79 and 80 or older.

^b^ Number of incident colon cancer cases.

^c^ Rates were adjusted for age and primary payer across these categories: 25-59, 60-69, 70-79 and 80 or older; and uninsured, Medicaid, Medicare and private insurance.

^d^ Rates were adjusted for age and neighborhood poverty across these categories: 25-59, 60-69, 70-79 and 80 or older; and < 5% poor, 5-29% poor and > 30% poor.

**TABLE 7S—Main Effects and Interactions of Neighborhood Poverty and Primary Payer by Gender on Receipt of Optimum Treatment^a^ Among Colon Cancer Patients with Stage III Disease: Rates and Rate Ratios**

______________________________________________________________________________

Women Men

No.^b^ Rate RR (95% CI) No.^b^ Rate RR (95% CI)

Independent main effects

Neighborhood poverty^c^

< 5% poor 133 .714 1.00 133 .656 1.00

5-29% poor 131 .573 **0.80** (0.67, 0.96) 115 .649 0.99 (0.69, 1.44)

> 30% poor 109 .582 **0.81** (0.67, 0.98) 103 .626 0.95 (0.78, 1.15)

Primary payer^c^

Uninsured or Medicaid 53 .684 1.00 42 .575 1.00

Medicare or private 320 .618 0.90 (0.71, 1.14) 309 .651 1.13 (0.89, 1.44)

Adjusted main effects

Neighborhood poverty^d^

< 5% poor 133 .710 1.00 133 .670 1.00

5-29% poor 131 .577 **0.81** (0.67, 0.98) 115 .649 0.97 (0.77, 1.22)

> 30% poor 109 .567 **0.80** (0.66, 0.97) 103 .635 0.95 (0.81, 1.12)

Primary payer^e^

Uninsured or Medicaid 53 .655 1.00 42 .627 1.00

Medicare or private 320 .610 0.93 (0.76, 1.14) 309 .648 1.03 (0.87, 1.22)

______________________________________________________________________________

Poverty by payer interaction among men

> 30% poor < 30% poor

No.^b^ Rate RR (95% CI) No.^b^ Rate RR (95% CI)

______________________________________________________________________________

Primary payer^c^

Uninsured or Medicaid 19 .413 1.00 23 .694 1.00

Medicare or private 84 .667 **1.62** (1.00, 2.61) 225 .645 0.93 (0.70, 1.24)

______________________________________________________________________________

Notes. RR = standardized rate ratio, CI = confidence interval. Confidence intervals were derived from the Mantel-Haenszel χ^2^ test. All adjustments were internal and direct, using this study’s population of colon cancer cases as the standard.

^a^ Optimum treatment: received colon cancer-directed surgery within 30 days of diagnosis and received adjuvant chemotherapy within 45 days of surgery.

^b^ Number of incident colon cancer cases.

^c^ Rates were age-adjusted across these categories: 25-64 and 65 or older.

^d^ Rates were adjusted for age and primary payer across these categories: 25-64 and 65 or older; and uninsured/Medicaid and Medicare/private insurance.

^e^ Rates were adjusted for age and neighborhood poverty across these categories: 25-64 and 65 or older; and < 5% poor, 5-29% poor and > 30% poor.
